# Effect of neoadjuvant chemotherapy on CD14 + CD16 + monocytes and soluble CD163 in Egyptian breast cancer patients

**DOI:** 10.1038/s41598-025-88719-5

**Published:** 2025-02-15

**Authors:** Sara Youssry, Hossam Ghoneim, Riham Barakat, Eman ElAlfy

**Affiliations:** 1 Department of Immunology and Allergy, Medical Research Institute, Alexandria University, Alexandria, Egypt; 2 Cancer Management and Research Department, Medical Research Institute, Alexandria University, Alexandria, Egypt

**Keywords:** CD14, CD16, Monocyte, sCD163, NACT, Breast cancer, Clinical response, Cancer, Immunology, Biomarkers, Medical research, Oncology

## Abstract

Neoadjuvant chemotherapy (NACT) influences the anticancer response by favourably altering the immune microenvironment. However, the effects of NACT on peripheral monocytes and their prognostic contribution to the NACT response have not yet been clarified. We aimed to evaluate the potential therapeutic responses and possible predictive value of double-positive (CD14 + CD16 +) monocytes and soluble CD163 (sCD163) in Egyptian breast cancer patients. Blood samples were obtained before and after neoadjuvant therapy from 30 patients with invasive breast cancer, and the expression of CD14 and CD16 was assessed via flow cytometry. The patients’ sCD163 levels were also determined in both the serum and culture supernatant using enzyme-linked immunosorbent assay (ELISA). The results revealed that NACT was associated with a significant decrease in double-positive monocytes and sCD163 levels. In addition, both double-positive monocytes and serum sCD163 were significantly associated with a partial clinical response. Double-positive monocytes and serum sCD163 levels may be related to therapeutic response, suggesting their possible predictive value in breast cancer patients receiving NACT.

## Introduction

Breast cancer is the most common cancer among women worldwide, with an estimated incidence of 2.3 million cases in 2020, which is predicted to reach 4.4 million in 2070^1^. It accounts for approximately 24.5% of all cancer cases and 15.5% of cancer deaths^2^. It is also the most prevalent malignancy among Egyptian women, representing 32.4% of new cancer cases^3^.

Breast cancer cells interact with various innate immune cells, including natural killer (NK) cells, neutrophils and monocytes/macrophages, that can regulate tumor cells proliferation, development and progression^4^. It has been reported that monocyte subpopulations can react to tumor presence and are associated with both tumor characteristics and treatment efficacy^5^. In addition, neoadjuvant chemotherapy (NACT) is known to have systemic immunologic effects in which chemotherapeutic agents can edit macrophages in tumor-protective or antitumor directions^6^. However, little is known about the role of different monocyte subpopulations and their prognostic contribution to chemotherapeutic agents in breast cancer.

Circulating monocytes are a heterogeneous population of cells that can be initially separated into two distinct phenotypic and functional subpopulations: CD14 + CD16- cells and CD14 + CD16 + cells^7^. CD14 + CD16 + monocytes are characterized by the capacity to produce proinflammatory cytokines, which are significantly increased under inflammatory conditions^8^. CD14 + CD16 + monocytes may be a useful indicator for the early diagnosis of breast cancer^9^.

CD163 is a monocyte/macrophage scavenger receptor that is highly expressed on tumor-associated macrophages (TAMs) and is considered a macrophage activation marker. Soluble CD163 (sCD163) is released into the serum or other body fluids after proteolysis of membrane proteins^10^. It has been reported that sCD163 levels are elevated in the serum of tumor patients and can be used to estimate the total body anti-inflammatory type 2 macrophage (M2) load. Moreover, serum sCD163 might be a novel prognostic marker in some cancers^11^; however, little is known about the clinical significance and prognostic value of sCD163 in breast cancer.

The current study aimed to evaluate alterations in double-positive (CD14 + CD16 +) monocytes and sCD163 levels in response to NACT in an attempt to reveal their potential prognostic value in Egyptian breast cancer patients.

## Subjects and methods

### Subjects

The current study included 30 patients with invasive breast cancer who were selected for NACT and 15 age-matched healthy females as a control group. Patients were recruited from the Cancer Management and Research Department, Medical Research Institute, Alexandria University. All patients were examined and reported for age and different clinicopathological parameters, including tumor type, stage, grade, lymph node involvement, vascular invasion and tumor metastasis, as well as estrogen receptor/progesterone receptor and human epidermal growth factor receptor 2 (HER2) status^12^. The exclusion criteria included patients with a history of any other cancer or any immune-mediated disease and those with equivocal histologic results. Venous blood samples (6 ml) were obtained from all subjects after providing written informed consent according to the rules approved by the medical ethical committee of the Medical Research Institute. The current study followed the principles outlined in the Declaration of Helsinki for the use of human subjects.

### Chemotherapy plan and study design

The standard neoadjuvant treatment regimen consisted of four cycles of adriamycin and cyclophosphamide (AC) (Adriamycin 60 mg/m2, cyclophosphamide 600 mg/m2) every 21 days followed by 12 weekly doses of paclitaxel (Taxol) (80 mg/m2). Blood samples were collected from patients prior to the first cycle of NACT (group 1; pre-NACT), after receiving 4 cycles of AC (group 2A; 4AC) and after finishing the last cycle of the NACT regimen (group 2B; 4AC + Taxol).

### Clinical response of the tumor to NACT

The clinical measurement of the tumor response to NACT was assessed by two independent experienced physicians by directly measuring the 2 axes of the tumor—the longest one and the longest perpendicular to it—through both physical examination and ultrasound measurements. We recorded the response according to the International Union Against Cancer criteria^13^. No clinical evidence of any palpable mass at the original site was considered a complete clinical response (cCR), while a partial clinical response (cPR) was defined as a 50% or greater reduction in the bidimensional tumor mass measurements. Assessment of response was performed after receiving 4 cycles of AC (group 2A; 4AC) and after finishing the last cycle of the NACT regimen (group 2B; 4AC + Taxol).

### Peripheral blood mononuclear cells (PBMCs) isolation

Heparinized venous blood samples were diluted and layered gently over Ficoll-Hypaque (1077) (Sigma‒Aldrich Chemical Company)^14^. After centrifugation for 30 min at 1800 rpm, the interface cells containing PBMCs were carefully aspirated, pelleted and resuspended in 1 ml of RPMI (1640) to determine cell count and viability using a hemocytometer and trypan blue (0.2%).

### Short-term culture of PBMCs:

PBMCs (2 × 10^6^ cells/ml) were dispensed in 24-well tissue culture plates for 24 h in the presence of the polyclonal monocyte activator lipopolysaccharide (LPS) (100 ng/ml)^15^. After incubation at 37°C in a 5% humidified CO2 incubator, the supernatants were collected and stored at -80°C for evaluation of the sCD163 concentration.

### Analysis of CD14 and CD16 surface expression

Freshly isolated PBMCs were stained using the following monoclonal antibodies: anti-human leukocyte antigen (HLA)-DR phycoerythrin (PE), anti-human CD14 fluorescein isothiocyanate (FITC) and anti-human CD16 phycoerythrin (PE). Monocytes were gated based on cell size and complexity. Cells were then gated in an HLA-DR/CD14 plot to exclude HLA-DR-negative natural killer cells, followed by analysing cells for CD14 and CD16 expression to determine double-positive monocyte subsets^16^. The expression of CD14 and CD16 was determined by two-color flow cytometry. The cells were tested on a BD FACS Calibur flow cytometer (FACS Calibur, Becton–Dickinson, USA) using Cell Quest software (Becton–Dickinson).

### Detection of sCD163 levels

The concentrations of sCD163 in both culture supernatants (induced sCD163) and serum before and after NACT intervention were determined using commercially available sandwich ELISA kits according to the manufacturer’s instructions (Bioassay Technology Laboratory)^17^.

### Statistical analysis

The values are expressed as the means ± SDs and were analysed using SPSS statistical software version 20.0. (Armonk, NY: IBM Corp.). Shapiro–Wilk test was applied to evaluate the normality of distribution. Mann‒Whitney test was applied to compare non normally distributed quantitative variables between two groups. Multiple comparisons were performed using one-way ANOVA, followed by post hoc test. The significance of the obtained results was judged at the 5% level.

## Results:

### Demographic and pathological data of the studied breast cancer patients

Thirty patients with invasive breast cancer were enrolled. The demographic and pathological characteristics of the studied patients are summarized in Table 1.

**Table 1:** Demographic and pathological data of the studied breast cancer patients.

Breast cancer patients (n = 30)	No	%
Age ≤ 45 > 45	12 18	40 60
Family history No Yes	26 4	86.7 13.3
Menopausal status No Yes	14 16	46.7 53.3
Stage		
II	10	33.3
III	20	66.7
Lymph nodes stage		
cN0	4	13.3
cN1	10	33.3
cN2	12	40
cN3	4	13.3
Nodal status		
0	4	13.3
1–2	18	60
> 2	8	26.7
Vascular Invasion		
No	20	66.7
Yes	10	33.3
Histopathological type		
Invasive ductal carcinoma (IDC)	26	86.7
Invasive lobular carcinoma (ILC)	1	3.3
Invasive micropapillary carcinoma (IMC)	3	10
Grade		
GII	28	93.3
GIII	2	6.7
Estrogen receptor (ER)		
Negative	7	23.3
Positive	23	76.7
Progesterone receptor (PR)		
Negative	10	33.3
Positive	20	66.7
Her2		
Negative	23	76.7
Positive	7	23.3

Her2: human epidermal growth factor receptor 2.

### Effect of NACT on peripheral blood biomarkers in breast cancer patients

We assessed the impact of NACT on specific leukocyte subpopulations, including the absolute neutrophil count (ANC), absolute lymphocyte count (ALC) and absolute monocyte count (AMC), obtained from CBC. As shown in Table 2, NACT was associated with significant alterations in the ANC, ALC and AMC in all patients. We found that the ANC and ALC decreased significantly during NACT, where post-NACT (groups 2A and 2B) showed a significant decrease in the means of ANC and ALC compared to pre-NACT (group 1). On the other hand, AMC levels increased after 4 cycles of AC (group 2A; 4AC) and decreased after paclitaxel (Taxol)-based treatment (group 2B; 4AC + Taxol) reaching the control level.

**Table 2:** Comparison of peripheral blood biomarkers among the different studied groups.

peripheral blood biomarkers	Breast cancer patients (n = 30)			Control (n = 15)
	Pre NACT	Post NACT		
		4AC	4AC + Taxol	
Total Leucocyte Count (TLC)				
Mean ± SD	8.17 ± 2.32	6.12 ± 1.96	4.07 ± 1.64	7.47 ± 0.66
Sig. bet. groups	p_1_ = 0.001*,p_2_ < 0.001*,p_3_ < 0.001*			
(p)	0.262	0.011*	< 0.001*	
Absolute Neutrophil Count (ANC)				
Mean ± SD	4.76 ± 1.75	3.75 ± 1.46	2.45 ± 1.0	4.73 ± 0.95
Sig. bet. groups	p_1_ = 0.003*,p_2_ < 0.001*,p_3_ = 0.003*			
(p)	0.630	0.013*	< 0.001*	
Absolute lymphocyte count (ALC)				
Mean ± SD	2.60 ± 0.77	1.70 ± 0.63	1.14 ± 0.39	2.32 ± 0.43
Sig. bet. groups	p_1_ = 0.001*,p_2_ < 0.001*,p_3_ < 0.001*			
(p)	0.048*	0.002*	< 0.001*	
absolute monocyte count (AMC)				
Mean ± SD	0.56 ± 0.11	0.63 ± 0.15	0.33 ± 0.09	0.36 ± 0.10
Sig. bet. groups	p_1_ < 0.001*,p_2_ < 0.001*,p_3_ < 0.001*			
(p)	< 0.001*	< 0.001*	0.358	

The data were assessed using the Mann‒Whitney test (U).

*Fr *Friedman test, Sig. bet. Groups were subjected to a post hoc test (Dunn’s test).

p: p value for comparisons between BC patients and controls in each group.

p_1_: p value for comparing Pre-NACT and Post-NACT (4AC).

p_2_: p value for comparing Pre-NACT and Post-NACT (4AC + TX).

p_3_: p value for comparing post-NACT (4AC) and post-NACT (4AC + TX).

*: Statistically significant at p ≤ 0.05.

### Effect of NACT on the double-positive CD14+ CD16+ monocyte subset in breast cancer patients

**Fig. 1:**
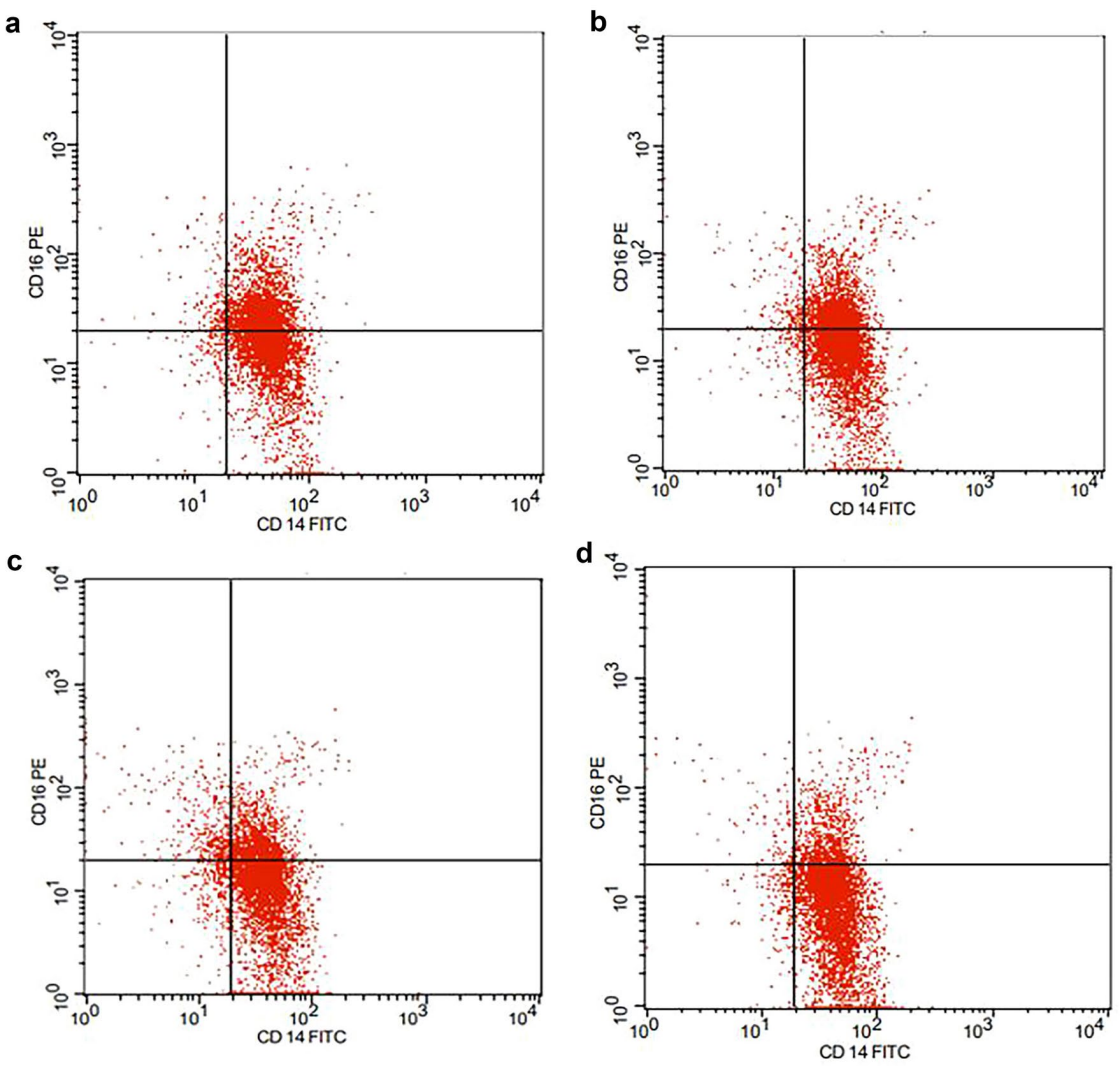
Frequencies of the CD14 + CD16 + monocyte subset in the studied groups. Monocytes were stained with anti-human CD14 fluorescein isothiocyanate (FITC) and anti-human CD16 phycoerythrin (PE). Representative dot plots of (**a**) pre-NACT (group 1), (**b**) post-NACT (4AC; group 2A), (**c**) post-NACT (4AC + Taxol; group 2B) and (**d**) healthy control samples are shown.

We evaluated the double-positive CD14 + CD16 + monocyte subset in which PBMCs were double-labelled with anti-CD14 and anti-CD16 conjugated antibodies. As shown in Table 3, the results showed that NACT application was associated with a marked decrease in the proportion of the CD14 + CD16 + monocyte subset in comparison to that in group 1 (pre-NACT), with a significant decrease in this subset in the paclitaxel (Taxol)-treated group (group 2B; 4AC + Taxol) compared to that in group 2A (4AC). Despite the ameliorative effects of NACT on this monocyte subset, its proportion was still significantly greater than that in the control group (p < 0.001) (Fig. 1).

**Table 3:** Comparison between different studied groups regarding different monocytic markers.

Monocytic markers	Breast cancer patients (n = 30)			Control (n = 15)
	Pre NACT	Post NACT		
		4AC	4AC + Taxol	
CD14 + CD16 +				
Mean ± SD	40.50 ± 7.84	32.23 ± 5.63	25.17 ± 6.76	13.27 ± 5.73
Sig. bet. periods	p_1_ < 0.001*,p_2_ < 0.001*,p_3_ = 0.008*			
(p)	< 0.001*	< 0.001*	< 0.001*	
Serum sCD163 (ng/ml)				
Mean ± SD	3.87 ± 1.07	2.98 ± 0.84	2.09 ± 0.51	0.78 ± 0.24
Sig. bet. periods	p_1_ < 0.001*,p_2_ < 0.001*,p_3_ < 0.001*			
(p)	< 0.001*	< 0.001*	< 0.001*	
Induced sCD163 (ng/ml)				
Mean ± SD	8.09 ± 1.75	6.44 ± 1.72	4.98 ± 1.57	2.68 ± 0.13
Sig. bet. periods	p_1_ < 0.001*,p_2_ < 0.001*,p_3_ = 0.001*			
(p)	< 0.001*	< 0.001*	< 0.001*	

The data were assessed using the Mann‒Whitney test (U).

p: p value for comparisons between BC patients and controls in each period.

Sig. bet. Pre, post-NACT (4AC) and post-NACT (4AC + TX) were performed using the post hoc test (Dunn’s) for the Friedman test.

p_1_: p value for comparing Pre-NACT and Post-NACT (4AC).

p_2_: p value for comparing Pre-NACT and Post-NACT (4AC + TX).

p_3_: p value for comparing post-NACT (4AC) and post-NACT (4AC + TX).

*: Statistically significant at p ≤ 0.05.

### Effect of NACT on sCD163 levels in breast cancer patients

**Fig. 2:**
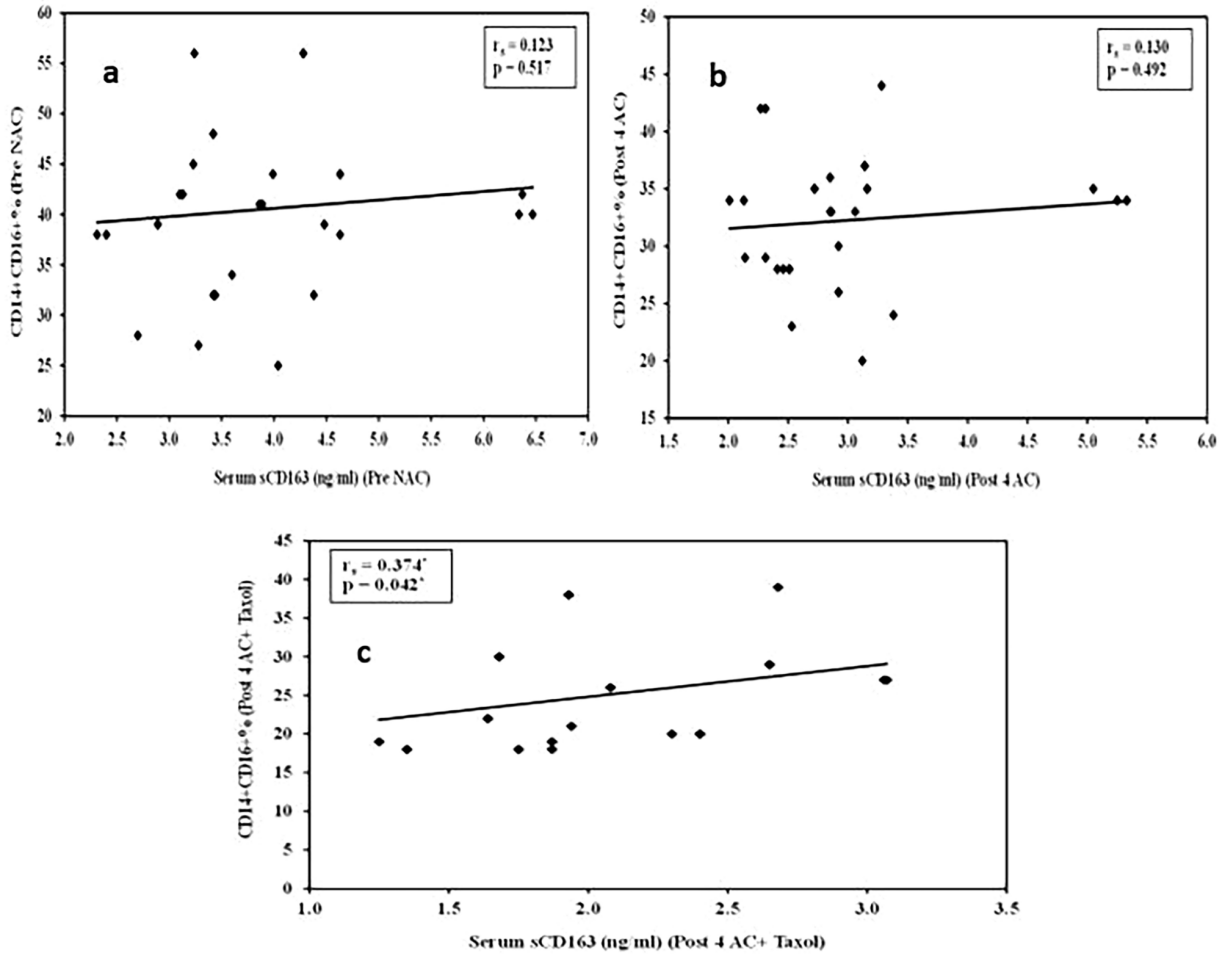
Correlation between CD14 + CD16 + % with serum sCD163 (ng/ml) in breast cancer patients. (**a**) Correlation analysis between the proportion of CD14 + CD16 + monocyte subset with serum sCD163 (ng/ml) in pre-NACT (group 1), (**b**) Correlation analysis between the proportion of CD14 + CD16 + monocyte subset with serum sCD163 (ng/ml) in post-NACT (4AC; group 2A) and (**c**) Correlation analysis between the proportion of CD14 + CD16 + monocyte subset with serum sCD163 (ng/ml) in post-NACT (4AC + Taxol; group 2B).

As shown in Table 3, the levels of sCD163 in both the serum and culture supernatant were assessed. The results revealed that both the serum and induced sCD163 levels were significantly greater in all patient groups than in the control group (p < 0.001). Our results also showed that the means of both the serum and induced sCD163 levels were significantly lower in the post-NACT groups than in the pre-NACT group. Furthermore, the serum sCD163 level was positively correlated with the proportion of the CD14 + CD16 + subset (rs = 0.374, p = 0.042) in group 2B (4AC + Taxol)) (Table 4) (Fig. 2).

**Table 4:** Correlation between the different studied monocytic markers in breast cancer patients (n = 30).

	Serum sCD163 (ng/ml)	
Pre NACT		
CD14 + CD16 +	**r**_**s**_	0.123
	**p**	0.517
Post NACT (4AC)		
CD14 + CD16 +	**r**_**s**_	0.130
	**p**	0.492
Post NACT (4AC + TX)		
CD14 + CD16 +	**r**_**s**_	0.374
	**p**	0.042*

r_s_: Spearman coefficient.

*: Statistically significant at p ≤ 0.05.

### Associations of double-positive monocytes and sCD163 levels with the clinical response to NACT

To elucidate the predictive value of the studied immunological parameters, we analysed the possible associations between these parameters and clinical response, which were classified as complete response (CR) or partial response (PR). Our results revealed that both the serum sCD163 level and the percentage of double-positive monocytes were significantly associated with a partial response to NACT (p = 0.005 and < 0.001, respectively) (Table 5).

**Table 5:** Relationship between clinical response and different monocytic markers in the post-NACT groups.

	Clinical response			
	Post NACT (4AC)		Post NACT (4AC + Taxol)	
	CR (n = 3)	PR (n = 27)	CR (n = 14)	PR (n = 16)
Serum sCD163 (ng/ml)				
Mean ± SD	2.46 ± 0.41	3.04 ± 0.86	1.81 ± 0.37	2.32 ± 0.51
Test of Sig. (p)	p = 0.200		p = 0.005*	
Induced sCD163 (ng/ml)				
Mean ± SD	7.10 ± 2.10	6.37 ± 1.70	4.57 ± 0.73	5.34 ± 2.01
Test of Sig. (p)	p = 0.350		p = 0.728	
CD14^+^CD16^+^				
Mean ± SD	29.33 ± 0.58	32.56 ± 5.85	19.43 ± 1.45	30.19 ± 5.38
Test of Sig. (p)	p = 0.387		p < 0.001*	

The data were assessed using the Mann‒Whitney test (U).

p: p value for the relationship between clinical response and different markers post-NAC.

*: Statistically significant at p ≤ 0.05.

## Discussion

Clinical complete response (cCR) is considered a surrogate endpoint for favourable survival in breast cancer patients treated with NACT^18^. It has been reported that immune infiltration in breast cancer predicts both a better prognosis and greater sensitivity to chemotherapy^19^. However, the dynamic changes in patients’ immune cells under NACT remain unclear.

The role of the innate immune system in tumor immune surveillance has long been recognized, and selective context-specific targeting of the innate immune system has the potential to become a cornerstone of immunotherapy strategies for the treatment of solid tumors^20^. However, there is still an urgent need to develop robust and inexpensive biomarkers in breast cancer owing to the presence of few predictive biomarkers for response to chemotherapy^21^.

The present study revealed that compared with 4 cycles of AC NACT, paclitaxel-based NACT was associated with a marked decrease in AMC. In concordance, it has been reported that an elevated AMC is associated with a poor prognosis in patients with solid cancer^22^, reflecting the observed decrease in the AMC with a complete treatment regimen of paclitaxel. However, the increase in AMC with cyclophosphamide may be attributed to the role of cyclophosphamide as a potent stimulator of innate immunity, where a possible positive interaction was shown between IFN-I, which may control monocyte abundance, and cyclophosphamide, which may potentiate antitumor immune responses^23^.

Human monocytes are a heterogeneous population and are divided into two subsets based on the expression of CD16. A considerable proportion of CD14 + CD16 + monocytes can be induced and expanded in breast cancer patients. Similarly, our results showed a significant increase in the frequency of the CD14 + CD16 + monocyte subset in all patient groups compared to that in healthy controls. Likewise, Patysheva et al. observed a greater count of CD163-expressing intermediate monocytes in breast cancer patients compared with healthy women^5^.

This upregulation may be attributed to that tumor cells produce different factors (such as IL‐10 and CCL2) that can modulate circulating monocytes^24^. In addition, regulatory DCs differentiated from CD14 + CD16 + monocytes can induce Th2 polarization, resulting in tumor immune tolerance^9^. Moreover, it has been demonstrated that CD14 + CD16 + CD81 + ITGAX + CSF1R + monocytes/macrophages secrete specific pro-fibrotic as well as pro-metastatic growth factors, including fibronectin 1, cathepsins (CTSB and CTSD) and osteopontin^25^. On the other hand, a marked decrease in monocytes was found in squamous cell carcinoma of the head and neck as well as in both cholangiocarcinoma and hepatocellular carcinoma before and after surgical procedures^26^. This discrepancy may be cancer type dependent.

More importantly, our results revealed a marked decrease in the frequency of the CD14 + CD16 + monocyte subset in post-NACT samples in which paclitaxel-based treatment had a markedly lower frequency than did treatment with group 2A (4AC). In line with these findings, chemotherapy was shown to exert its effects via elimination of the CD14 + CD16 +  + pro-inflammatory cell subset^27^.

Integrated investigations of the interplay between cells of the immune system and cancer are expected to enhance cancer diagnoses and treatment in the future^28^. It has been reported that CD163 may be released from tissue macrophages and monocytes by a metalloprotease-dependent shedding pathway, involving the inflammation-inducible enzyme TNF-α converting enzyme (TACE/ADAM17)^29^. Consequently, sCD163 has been suggested as a surrogate marker of TNF-α, pointing towards sCD163 as a possible inflammatory mediator^30^. High levels of sCD163 have been associated with disease progression and clinical outcome in different cancer types^31^ and may be a biomarker for predicting the tumor response to different therapies^32,33^. However, little is known about the prognostic value of sCD163 in breast cancer.

The present study showed that both the serum and induced sCD163 levels were significantly greater in all patient groups than in the healthy control group and were positively correlated with the CD14 + CD16 + monocyte subset. Additionally, compared with pre-NACT samples, post-NACT samples showed a significant decrease in sCD163 levels, and this reduction was more significant in the paclitaxel-treated group than in the 2A (4AC) group. In agreement with these findings, it has been reported that sCD163 levels are greater in cancer patients than in healthy people and that sCD163 levels may decrease after treatment^11^. This may be related to the fact that the levels of sCD163 are affected by the upregulation of CD163 surface expression. Moreover, it has been demonstrated that TNF-α, a powerful pro-cancer cytokine, induces shedding of cell surface molecules from tumor cells^34^. Furthermore, paclitaxel was shown to reduce the levels of CD163 and IL-10 in human primary PBMCs^35^.

It can be concluded from the above results that double-positive monocytes and serum sCD163 levels may have a negative prognostic value in response to NACT, highlighting their future importance as potential indicators to guide NACT in breast cancer patients.

## Data Availability

The datasets generated and/or analyzed during the current study are not publicly available [to protect study participant privacy] but are available from the corresponding author on reasonable request.

## Abbreviations

NACT: Neoadjuvant chemotherapy
sCD163: Soluble CD163
AC: Adriamycin and cyclophosphamide
PBMCs: Peripheral blood mononuclear cells
FITC: Fluorescein isothiocyanate
PE: Phycoerythrin
ANC: Absolute neutrophil count
ALC: Absolute lymphocyte count
AMC: Absolute monocyte count
CR: Complete response
PR: Partial response
